# Paralysis*Read before the New York State Veterinary Medical Association.

**Published:** 1892-02

**Authors:** J. M. Chase


### PARALYSIS.*

By Dr. J. M. Chase.

Paralysis is a partial or complete cessation of muscular contraction caused by diminution or complete loss of the conducting power or stimulation of the motor nerves. Paralysis may be divided into partial or general. And partial into two classes, Hemiplegia and Paraplegia and where small parts of the body such as tail or face might come under the head of partial or local paralysis. As general paralysis cannot occur without producing immediate death it will not be necessary to make any deductions in regard to it, but with hemiplegia and paraplegia the practitioner often comes in contact, and it is principally with these that I shall have to deal in this paper. Hemiplegia, or paralysis of one side or half of the body is not met with in our patients to the extent that it is in the human family, because our subjects are not as subject to brain disorders and mental strain and activity. The causes of hemiplegia are principally, tumors in the brain particularly of the lateral ventricles, softening of one hemisphere of the cerebrum, pressure from extravasated blood clots or thrombi, fracture of cranium, but generally is the result of emboli obstructing one or more blood vessels in the brain. When it is the result of a disease of the brain which has existed prior to the paralysis particularly if of an imflamatory character, it seldom attacks the whole of one side of the body, but is confined to one side of head or neck or one limb, and may in such cases pass off in a short time. Sensibility in most cases is not impaired, but in some cases the sensory as well as motor nerves are involved.

Having given the principal causes in our subjects, we will direct your attention to the symptoms and treatment. In hemiplegia the attack may be very sudden, the animal falling down powerless to move one side of the body. The side that is affected presents the following conditions : the ear, part of lips will be relaxed, tongue may hang out of mouth, tail curved side ways, often accompanied by inability to swallow, together with a constant flow of urine as fast as secreted. The sensibility is usually lost as well as motion, but not always. Coldness of the limbs affected is a usual sign, though they may be warmer than natural. The

* Read before the New York State Veterinary Medical Association.

prognosis in severe cases is unfavorable, but in slight attacks when the animal remains in a standing position the recovery is quite probable under proper treatment.

Next we come to another form of paralysis of the posterior parts of the body. Under this head of paraplegia come spinal meningitis and myelitis. Paralysis of the posterior extremities is usually due to some injury or inflamation affecting the spinal cord. Symptoms of paraplegia are easily recognized by a weakness and imperfect control of the hind legs and powerless tail, and the passage of urine and fæces is done without any voluntary action, showing a loss of nerve force to the parts posterior to the seat of injury or disease. There are also likely to form large ulcers and sores on hips and sides of body which weaken the animal very rapidly and hasten final dissolution.

Next I shall give a short account of the causes, symptoms, pathology and treatment of spinal meningitis as it occurs in our patients. It is due to irritant properties of blood poisons, exhaustion or exposure, spinal concussions or injuries of any kind to the spine. Tumors, caries of the vertebræ, rheumatism, and it may be in the form of an epidemic. The usual symptoms are ushered in by a chill, a rise of temperature, general weakness and a disposition to be uneasy and shift positions of legs. Soon a painful convulsive twitching of the muscles sets in followed by muscular rigidity along the spine, and upon attempting to move the animal stiffens and great pain will be evinced. Retention or incontinence of urine and often sexual excitement is present. The characteristic symptoms of spinal meningitis are a high fever and spinal symptoms in the early stages of the disease, which usually become chronic after a time. Pathology, there will be an effusion of serum between the membranes of the cord which becomes plastic and adheres to the pia-mater which serves to maintain a state of paralysis for a long time after the acute symptoms have passed off. Finally, atrophy, softening and even abscess may develop within the cord.

*Treatment.*—Bags filled with ice should be applied along the spine to be followed later with strong blisters. The fever should be controlled with antifebrine, aconite or Norwoods tincture of veratrium viridi in 20 min doses every hour together with doses of extract of belladonna every 6 hours until the pupils of the eyes become dilated. If there is a great amount of pain hypodermic injections of morphine should be given to relieve the intense suffering which marks spinal diseases. The animal should be kept as quiet as possible, and free from excitement. The urine when there is retention must be drawn every 5 or six hours. Placing in slings is often beneficial, and care must be taken to empty the bladder before attempting to raise the animal with the slings, as the bladder when very much distended, is apt to be ruptured on applying so much force to the abdominal walls. Iodide of potassium nux Vomica and the Iodide of Iron are indicated in the convalescent stages, and electricity may be advantageously used to the affected muscles.

*Myelitis*, inflammation of the substance of the cord. This disease is not usual in our patients, except as a sequel of spinal meningitis, and is to be treated in the same way as this disease, but it is not usual for a recovery to be effected.

Under this head of paralysis comes a number of local lesions and affections of the nerves, such as locomotor ataxia, where there is a want of coördination of movements of the muscles of one or more limbs.

When confined to the posterior limbs it may be classed as a form of paraplegia, and is to be treated as such. Facial paralysis is met with in some cases and is manifested by pendulous lips, flaccid cheeks, and inability to grasp food, this is best treated by a sharp blister over seat of trouble. Paralysis of the intestines is a disease with which the practitioner may meet, and can only be diagnosed by negative symptoms, as when no results from cathartics can be obtained and absence of any signs of impaction and indigestion, may be caused by sudden checking of perspiration and to brain affections, or to reflex paralysis. Paralysis of the bladder is met with and is to be decided upon much in the same way as that of intestines, by negative rather than affirmative signs. Amaurosis is a disease which we often meet with, and one with which every practitioner ought to be very familiar, particularly in examination as to soundness. The eye looks natural on casual examination, but when examined carefully the affection is easily discovered ; inasmuch as no medical treatment can be applied to the optic nerve amaurosis may be properly classed among the forms of paralysis that are not amenable to treatment.

In summing up the treatment of paralysis, the general treatment consists in cold applications to spine with febrifuges to counteract the febrile symptoms, and in convalescent stage blisters to spine and nux vomica and iodide of potassium.

